# Consumers purchase intention in live-streaming e-commerce: A consumption value perspective and the role of streamer popularity

**DOI:** 10.1371/journal.pone.0296339

**Published:** 2024-02-15

**Authors:** Qi Zhang, Yuling Wang, Shaizatulaqma Kamalul Ariffin

**Affiliations:** 1 School of Foreign Languages and Literatures, Chongqing University of Education, Chongqing, China; 2 School of Languages, Literacies and Translation, Universiti Sains Malaysia, Penang, Malaysia; 3 Graduate School of Business, Universiti Sains Malaysia, Penang, Malaysia; Universidad Del Rosario, COLOMBIA

## Abstract

The rapid development of live-streaming e-commerce has driven billions of sales revenues and made customers’ purchase intention a life-and-death issue for sellers. This study examines the influencing factors of customers’ purchase intention from a value perspective by adopting and extending the Theory of Consumption Values (TCV). We also incorporated streamer popularity as a moderating variable to reveal its significant impact on live-streaming e-commerce. This study collected 457 valid online questionnaires from Chinese live-streaming e-commerce users. Our findings show that five of six consumption values, namely functional, social, emotional, conditional, and self-gratification value, are significant drivers of purchase intention. In addition, streamer popularity has strengthened the influence of functional, social, emotional, and self-gratification value on purchase intention. This study deepens the current understanding of live-streaming and customer value research by establishing and validating a comprehensive research model, and reveals the decisive role of multi-dimensional value and streamer popularity in live-streaming industry. The research findings could guide live-streaming merchants to increase sales by reallocating their resources to different consumption values and optimising their investment strategy in popular streamers.

## 1. Introduction

In recent years, live-streaming e-commerce has become a global economic and social trend that attracted growing attention. Since Walmart live-streamed its first shopping events on TikTok in December 2020, more and more retailers have partnered with platforms like Twitter, YouTube, and Facebook to hold live-streaming shopping events. In China, live-streaming e-commerce has also been adopted by leading e-commerce platforms such as Taobao and Jingdong, or content-sharing platforms such as Douyin and Kuaishou, creating an industry worth more than 200 billion US dollars [1].

Live-streaming e-commerce integrates the online shopping process into a live-streaming context [2]. It is a hybrid of three salient functionalities of video content, real-time communication, and consumption [3,4]. Products exhibited in live-streaming are much more vivid and realistic than the traditional campaign through texts or pictures [5]. Viewers can have a closer look at the products and listen to the streamer introduce how the product works, feels or smells. They can also acquire a direct impression by watching how the product looks or works on the streamer [6]. In addition, live-streaming e-commerce has a real-time communication function [7]. Users can bring up questions or demands and will be immediately responded by streamers [8,9]. Recently, more and more celebrities have been invited to the broadcasting room to be the streamer and help to promote the products. Celebrity streamers’ participation has further attracted millions of fans’ attention and drives billions of sales revenue [10]. With all these advantages that come with live streaming e-commerce, consumers’ purchase intention has been greatly stimulated [1,11]. Based on the report of Qianzhan industrial research institute [12], the total sales revenue of the Chinese live-streaming e-commerce industry has reached 195.2 billion US dollars by the end of the year 2020, and Taobao Live recorded a 24-hour sales revenue of 6.34 billion US dollars during 2020 Singles Day Shopping Festival (China’s Black Friday) [13].

Along with the prosperity of the overall live-streaming industry, some problems could be seen behind the shining revenue numbers. A growing Matthew effect is emerging in the unbalanced performance among different platforms and streamers [1]. For example, in 2020, the total sales revenue of the top three live-streaming platforms, Taobao, Kuaishou, and Douyin, has reached 140 billion US dollars, taking up more than 70% of the entire industry. Meanwhile, the ten leading streamers have occupied 5% of the industry’s revenue by creating 10 billion in sales [14]. Figure out how consumers’ purchase intentions are formed would be conducive to the sustainable and healthy development of the entire live-streaming industry. Besides the growing unbalanced development, there is also an imminent problem of merchants’ blind pursuit of popular streamers. To attract viewers and stimulate sales, an increasing number of celebrities and popular streamers are paid an astronomic commission for promoting products in live-streaming. Some of these endorsements have yielded worthy results, such as the most popular streamer on Taobao, Austin, has driven over 100 billion US dollars sales in 2022 [15]. On the contrary, some celebrities, despite their popularity and possession of millions of fans, are struggling with a poor sales record in live streaming e-commerce. Therefore, it is reasonable to ask how consumers’ purchase intention is formed in live-streaming e-commerce. Why do consumers choose certain live-streaming merchants over others? Will the streamer’s popularity influence the forming process of viewers’ purchase intention? If so, through what mechanism will it exert an influence?

Despite the growing popularity of live-streaming e-commerce, it has so far received only limited attention in previous literature [3,16]. Existing studies have repeatedly employed the uses and gratification theory [17], affordance theory [18] or information success model [19] to examine users’ behavioural outcomes, such as engagement [11,20], continued intention [16] or purchase intention [21,22]. However, most of these studies only consider external factors that influence users’ behaviour, such as technical features, streamer characteristics or live-streaming content [1,18,23]. Studies that focus on the internal value-based perspective are relatively scarce [23]. Moreover, customer value studies in live-streaming only involve generic value dimensions, such as utilitarian value [11], hedonic value [1], or social value [16], without look into other value perspectives that have significant potential influence in the live-streaming context. Therefore, customers’ perceived value in this specific research context remains unclear and requires deeper investigation [16].

Meanwhile, there is a trending phenomenon that an increasing number of popular celebrities have been invited as streamers [24], but not all celebrity streamers’ contributions to product sales are worth their sky-high commission. It is still unclear how exactly these popular streamers influence viewers’ decision-making process. Although celebrity endorsement has certain benefits, including increased customer attention, engagement or a more favourable attitude [25], previous studies on celebrity influence reveal that some famous celebrities, such as Britney Spears and Paris Hilton, are making some consumers buy less of the endorsed product [26]. Despite the complex nature of the problem, most prior studies in live-streaming have neglected celebrity endorser’s impact on consumer behaviour. The underlying logic distinguishing viewers to cultivate different levels of purchase intention when provided with similar values remains unclear. Our study attempts to fill these gaps by examining purchase intention from a viewer’s value perspective, also adopting streamer popularity as a moderator to uncover its influencing mechanism on viewers’ purchase intention.

This study could enrich the extant live-streaming literature by exploring the determinants of viewers’ purchase intention based on a multi-faceted value perspective. Specifically, this study adopts and extends the Theory of Consumption Values (TCV) by introducing self-gratification value as a new independent variable, which expands and increases its theoretical solidarity in this specific context. On the other hand, given that most existing live-streaming studies have neglected possible variations in celebrity streamers when examining the influence of users’ value perceptions on their behaviour, this study enriches the existing literature by employing streamer popularity as a moderator to examine it as a boundary condition in driving purchase intention. Our research findings would help merchants and live-streaming platforms realise which consumption values are most decisive in driving customers’ purchase intention and how streamers’ popularity affects this process so that the merchants and platforms can effectively boost sales by positioning these values in their marketing campaigns and investing in celebrity endorsers more wisely under the guidance provided by this study.

The remaining of this study is organized as follows. Section 2 presents a comprehensive literature review of our topic. Section 3 proposes a research model with hypotheses. Section 4 introduces the research method, and Section 5 presents the empirical study results. Finally, section 6 presents the discussions and implications with limitations and suggestions for future research.

## 2. Literature review

### 2.1 Live streaming e-commerce

Live streaming e-commerce refers to a new selling method that enables the sellers to directly communicate and promote to shoppers through online live product demonstrations, product promotional activities, and real-time shopping guidance [27]. In comparison to traditional e-commerce, it integrates the live-streaming advantages of enhanced visibility, authenticity, and real-time communications into the online shopping environment, thus enormously stimulating consumers’ buying behaviour [28]. We have summarized the recent studies on live-streaming e-commerce in Table 1. Most previous studies have been conducted to explore the features of live-streaming that would drive customers’ usage behaviour [18,29]. Several distinctive characteristics of live-streaming have been identified, such as immersion [18,30], interactivity [31,32] or design features [30,33]. Comparatively, only limited studies have considered the value-based constructs that would lead to viewers’ behaviour [23]. Stimulated by these features, there are mainly two behavioural outcomes of live-streaming e-commerce customers that have been discussed in prior studies. One of them is consumers’ usage intention. Chen and Lin [34] identified that flow, entertainment, social interaction, and endorsement will motivate consumers’ usage intention in live-streaming service. Similarly, Sjöblom and Hamari [35] revealed that viewers’ usage intention is driven by social connection, emotional factors, and stress release. Customer engagement is another widely discussed outcome of live-streaming e-commerce [36]. Xue et al. [20] found that live interactions would drive customer engagement through increased perceived usefulness and decreased risk. Continuing to examine customer engagement, Hu and Chaudhry [6] demonstrated a tight connection between relational bonds and live-streaming users’ engagement.

**Table 1:** Empirical studies on live-streaming e-commerce.

Authors	Theory		Antecedents		Outcomes			Involving Streamer Popularity
Yu et al. [37]	-		Viewer engagement		Gift consumption			N/A
Sun et al. [18]	IT Affordance Theory		Visibility; Metavoicing; Guidance shopping		Purchase intention			N/A
Cai and Wohn [31]	Uses & Gratification Theory		Interaction enjoyment; Substitutability of personal examination; Need for community; Trend Setting		Watching; Purchase			N/A
Wongkitrungrueng and Assarut [11]	Perceived Value Theory		Utilitarian value; Hedonic value; Symbolic value		Customer engagement			N/A
Park and Lin [38]	Fit theory		Wanghong-product fit; Live content-product fit; Self-product fit		Intention to buy			N/A
Xu [28]	SOR Framework		Streamer attractiveness; Para-social interaction; Information quality		Consumption; Social sharing			N/A
Hu and Chaudhry [6]	Relationship Marketing Theory		Financial bonds; Social bonds; Structural bonds		Consumer engagement			N/A
Xue et al. [20]	SOR Framework		Personalisation; Responsiveness; Entertainment; Mutuality; Perceived control		Social commerce engagement			N/A
Quan et al. [39]	Para-social Interactions Theory, Attractiveness Theory		Relationship rewards; Self-disclosure; Affective interactivity; Informative interactivity; Amount of information provided		Purchase intention			N/A
Hou et al. [3]	Uses & Gratification Theory		Interactivity; Social status display		Continuous watching intention; Consumption intention			N/A
Ma [19]	Information System Success Model		Information quality; Service quality; Argument quality		Satisfaction			N/A
Ma [40]	Uses & Gratification Theory		Perceived enjoyment; Perceived social interaction; Social presence; Perceived utility; Self-presentation		Live-stream shopping intentions			N/A
Singh et al. [16]	Perceived Value Theory		Effort expectancy; Performance expectancy; Convenience value; Monetary value; Emotional value; Social value		Continued intention			N/A
Li and Peng [41]	Attachment and Flow Theory		Trustworthiness; Entertainment		Gift-giving intention			N/A
Meng et al. [42]	Emotional contagion theory		Pleasant; Arousal; Emotional trust; Admiration		Purchase intention			N/A
Kang et al. [43]	SOR Framework		Interactivity (responsiveness, personalization)		Customer engagement			N/A
Li et al. [44]	Socio-technical theory, Attachment Theory		Interaction; Identification; Synchronicity; Vicarious expression		Visit duration; User retention			N/A
Lu and Chen [45]	Uncertainty reduction		Physical characteristic similarity; Value similarity		Purchase intention			N/A
Xu et al. [46]	Information asymmetry theory, Parasocial relationship theory		Centricity; Professionalism; Commitment; Reciprocity		Purchase intention			N/A
Guo et al. [1]	-		Beauty; Warmth; Expertise; Humor; Passion		Watching intention; Purchase intention			Yes
Zhang et al. [47]	Socio-technical theory		Active control; Synchronicity; Two-way communication; Personalisation; Visibility		Continuance intention			N/A
Liu et al. [48]	Motivation theory		Socializing; Media engagement; Remuneration; Product examination; Relaxation; Entertainment; Self-development; Immersion		Behaviour intentions; User satisfaction			N/A
Chen and Liao [5]	Social presence theory		Sense of community; Emotional support; Interactivity		Watching intention			N/A
Zheng et al. [49]	Flow theory		Social presence; Interactivity; Attractiveness; Expertise		Continuous watching intention; Purchase intention			N/A
Liao et al. [7]	Parasocial interaction theory, Flow theory		Communication style; Immersion; Parasocial interaction		Purchase intention			
This study	Theory of Consumption Values		Functional value, social value, emotional value, epistemic value, conditional value, self-gratification value		Purchase intention			Yes

In this study, we consider viewers’ purchasing intention as the primary concern and ultimate target desired by live-streaming e-commerce sellers [49], since profitability can only be achieved through sales performance [50]. Although a few studies have shed light on the purchase intention in live-streaming e-commerce, most of them are either tend to explain viewers’ purchase intention through technical features, such as interactive functions [37], enhanced product presentation [51] and IT affordances [18], or try to predict purchase intention from the streamer’s standpoint, such as streamers’ appeal [3], streamers’ characteristics [1], or streamer-product fitness [52]. Limited studies have tried to explain purchase intention from the viewer’s perspective. This study differed from previous research by arguing that viewers are the primary subject of purchase intention, and it is the viewers who will make the final purchase decision in live-streaming shopping. Therefore, this study attempts to approach purchase intention from the viewers’ standpoint and examine the values that would drive their purchase intention.

### 2.2 Customer perceived value and theory of consumption values

#### Customer perceived value

The concept of customer perceived value has always played a central role in customer behavioural studies [53,54]. It is usually described as a trade-off between quality and price by empirical researchers [55]. Zeithaml [56] brought up the most commonly used and widely spread definition of perceived value as users’ evaluation of a product’s performance based on their assessment of what is gained and what is given.

The concept of value has been widely recognised as the foundation of plentiful theories and disciplines in human cognition and behaviour [57]. Value could influence human behaviour [58], as people utilize values to decide and rationalize their actions [59]. Thus in the business field, the concept of value is also deemed as the basis for differentiating and guiding consumer behaviour [60] and plays a vital role in marketing and consumer behaviour studies [61]. It has been used to explain the motivation of customers’ choice behaviour [62], behavioural intentions [63,64], purchase intentions [65,66], adoption behaviour [67,68] and continuance behaviour [69,70]. More recently, value has also been depicted as the basis for differentiating companies from competitors and the key to establishing sustainable competitive advantage [71].

There are two major approaches to conceptualizing perceived value in previous literature: the uni-dimensional and the multi-dimensional approach [72,73]. The uni-dimensional approach regards value as an essentially utilitarian concept, which is formed based on the price and benefits that are eventually received in terms of functionality [74]. However, this approach is accused of simplifying the consumer’s perception of value by considering just price and quality [75]. Therefore, the inability to capture the comprehensive essence of perceived value leads to the inability to gain a competitive advantage [73,76]. Moreover, this approach has neglected the distinctive invisible, innate, and affective factors of the perceived value [77]. Therefore, compared with the uni-dimensional approach, more studies tried to catch the essence of value using a multi-dimensional approach [75,78–81]. Accordingly, this study will adopt a multi-dimensional value approach to apprehend customer perceived value in a live-streaming context.

#### Theory of consumption values

The theory of consumption values (TCV) was brought up by Sheth et al. [79] to explicate a basic question in the marketing area of how customers choose among different products or services. It proposes that consumers’ decision is made based on the integrated functioning process of five different consumption values. In the meantime, every single value has a separate and varying impact under a particular circumstance [79]. The publication of TCV has offered a useful tool for the following researchers in customer value [82,83]. It has been successfully adopted and expanded to various contexts to theorise consumers’ behaviour, including information systems [83,84], green products [85,86], mobile-based services [87] or tourism [74,81]. In the live-streaming background, Singh et al. [16] adopted TCV to assess the determinants that drive the continued use of live-streaming service, and they defined perceived value as the live-streaming service’s overall utility value that a customer perceives based on the cost-benefit trade-off. Their research proposed four dimensions to measure perceived value and found that perceived value is a prominent driver of customers’ continued intention. In the digital media context, Chakraborty et al. [88] examined users’ repurchase intention by adopting TCV. Their empirical study identified five values to have a significant influence on building trust and repurchase intention. Continuing to adopt the value theory, Wongkitrungrueng and Assarut [11] examined the influence of perceived value on customer engagement. Their study revealed that utilitarian, hedonic and symbolic value are positively related to trust and engagement in live-streaming social commerce sellers.

Although those prior live-streaming research have discussed the importance of perceived value in motivating consumers’ behaviour [36], our study takes the analysis further by considering extra contextual value dimension and boundary condition that might affect the value-behaviour relationship. A more comprehensive multi-dimensional value perspective would provide a more robust measurement of customers’ evaluation in explaining their purchasing behaviour [11,62], and it is considered appropriate in the live-streaming e-commerce context for several reasons: First, Viewers choose to buy from live-streaming due to multiple perceived values [16]. For instance, viewers could acquire functional value from vivid live product exhibition [1]; Emotional and social value could be perceived after interaction with popular streamers and other audiences [89]; Epistemic value is well received when trending information about new products are introduced, and conditional value could be influential when viewers get the best discount for buying the products [48]. Second, as a nascent business phenomenon, live-streaming e-commerce could provide customers with an unprecedented shopping experience by infusing entertaining and immersive factors into the shopping process [18,90]. Viewers are motivated to watch and buy from live-streaming e-commerce to reduce their tension and maintain inner equilibrium, also known as self-gratification value [91,92]. Therefore, generic value dimensions adopted in previous studies, such as utilitarian, hedonic or social value [1,11], might be inadequate to capture live-streaming customers’ value perception. Third, a value-based study is needed in China since consumers from middle-income countries tend to compare the values after using a service and drive their decisions [16]. Although several live-streaming studies have been conducted in China to examine consumer behaviour [18,22,44], few of them adopted a multi-dimensional value perspective to study viewers’ purchase intention. Based on these discussions, it is necessary to explain customers’ behaviour in live-streaming e-commerce based on a more comprehensive multi-faceted value perspective, and examine what values will eventually lead to viewers’ purchase intention [36,62].

### 2.3 Streamer popularity in live streaming e-commerce

Celebrity endorsement has long been recognized as an effective promotional strategy in the business world [93,94]. Previous studies have found that celebrity endorsement would profoundly influence consumers’ behaviour by transferring their preconceived positive image of a celebrity endorser to the endorsed product or brand [25]. Extant literature indicates that one in four advertisements is with celebrity endorsers [95,96]. Traditionally, Celebrity endorsers are referred to as a group of people who are publicly known because of their achievements in certain fields, usually in the category of famous actors, athletes, models, or singers [97]. Nowadays, driven by the lightening expansion of information technology and social media networks, consumers are more likely to be exposed to new types of celebrities or influencers [98], and the definition of a celebrity has been expanded [25]. “Ordinary” people are becoming “online” celebrities or “internet” micro-celebrities [99] and reach millions of followers on various platforms [98]. Various online celebrities have developed increasing social impacts on their large number of followers [100,101].

Nowadays, Live-streaming merchants are trying to make use of these celebrities’ influence by paying a large amount of money to invite them to join or host the online streaming show and promote products [102,103]. In this way, these celebrity streamers are becoming both product consumers and advertisers. They can promote the product to their followers in an instant two-way interactive environment, making the endorsement more natural and reliable [104]. With the advantages of both popularity and technology, celebrity streamers in e-commerce are more influential, persuasive, and powerful in influencing consumers’ behaviours [98]. As a result, online celebrities’ participation in live-streaming e-commerce has significantly stimulated sales and boosted the growth of the whole industry [25,105].

The streamer’s popularity refers to their ability to attract people’s preference to watch their online streaming shows [1]. It is usually reflected by their followers’ size and it is the key source of streamers’ competence in live-streaming e-commerce [98]. Streamers can be classified from nano endorsers with hundreds of followers to mega endorsers with millions of followers [106]. Previous studies indicate that endorsers with greater popularity usually lead to a larger influence on consumers’ behaviour [106]. Such as De Veirman et al. [107] revealed that users react more positively to Instagram influencers who have a larger number of followers; Jin and Phua [101] also corroborated that customers are inclined to choose products promoted by those endorsers with a higher number of followers on Twitter. These findings intuitively sound reasonable because people like to consider the number of followers as a decisive parameter in assessing advertising information [106,108]. This linear relationship between popularity and influence is also consistent with the logic of the popularity principle, which claims that the more followers an endorser has, the wider a message would reach, and a larger impact on consumers would be generated [106]. However, the impact of celebrity popularity on consumers’ behaviour may not be as straightforward as it seems and still needs to be further clarified [106,108]. A public research conducted in Dutch indicates that product endorsers who run in a small but accurate way are more likely to win customers’ trust [109]. In the live-streaming context, several studies have also found that streamer popularity is unrelated to their advertising outcomes [110,111]. Moreover, based on the 2020 Live-Streaming E-commerce Industry Report [14], there is no clear evidence that supports the positive connection between follower size and viewers’ purchase intention. Based on these mixed findings, this study attempts to find out how streamers’ popularity would interact with the influence of value on consumers’ behaviours by proposing it as a moderating variable.

## 3. Research model and hypothesis

**Fig 1:**
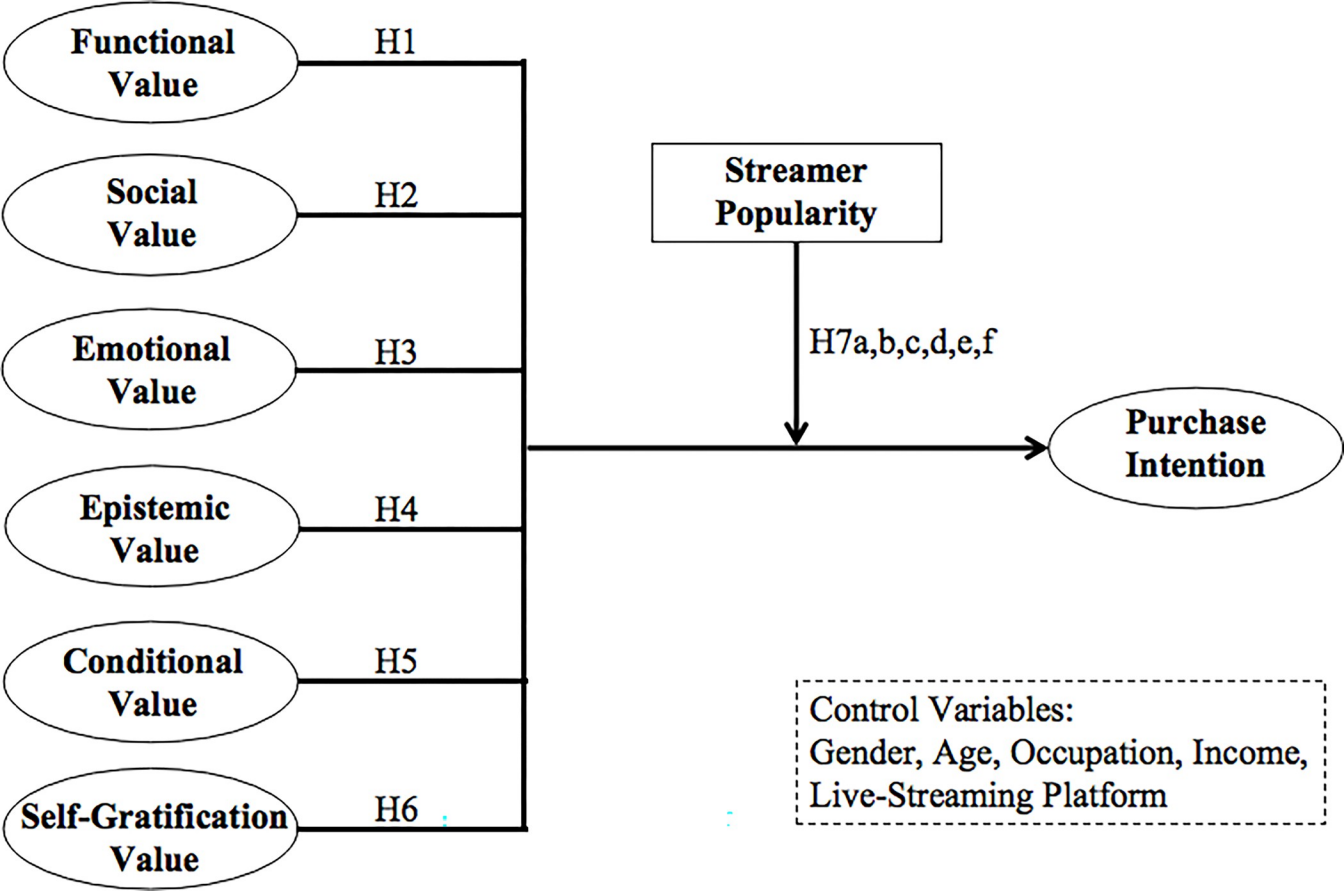
Theoretical framework.

The research framework is illustrated in Fig 1. This model presents a multi-dimensional value concept to examine the influencing factors of customers’ purchase intention in the live-streaming e-commerce context. Streamer popularity is incorporated to explore its influence on the forming process of customer purchase intention. The research model consists of six independent consumption value variables (functional, social, emotional, epistemic, conditional, and self-gratification value), a dependent variable purchase intention, a moderating variable streamer popularity and five control variables of gender, age, occupation, income and live-streaming platform.

### 3.1 Functional value and purchase intention

Functional value in TCV is described as a product or service’s value that is related to its salient functional attributes and is reflected in its physical performance [83]. In live-streaming, functional value is acquired from its salient functionalities, such as the lively exhibition of the product, real-time response to customers’ requests, and a convenient check-out system [6,9]. Functional value is found to be a crucial consumption value that drives consumers’ behaviour in various IS contexts [112], such as online shopping [113,114], mobile banking [82] or mobile application [115]. In live-streaming e-commerce, Yu and Zheng [36] suggested that a real-time exhibition of jewellery’s wearing effects could help make customers convinced by its functional performance, which significantly drives their purchase behaviour. Guo et al. [116] also demonstrated that an abundant amount of product-related knowledge, information and hands-on usage experience would be transferred to viewers in the live-streaming environment, thus allowing them to perceive higher functional value and drive their purchase intention. Similarly, Zhang et al. [117] found that strengthened knowledge sharing quality would positively influence customers’ sense of virtual community and increase customer-brand relationship. Based on the preceding discussions and literature, we postulate:

H1: Functional value positively influences customers’ purchase intention in live-streaming e-commerce.

### 3.2 Social value and purchase intention

Social value denotes a merchandise’s utility in improving users’ image among their social connections and networks [79,118]. Live-streaming e-commerce provides a social platform where the viewers can share their ideas, reviews or feedbacks to guide their purchase decision [16]. The viewers in the same broadcasting room serve as a social reference group and potentially persuade its members to purchase or behave according to some latent standards established among them [115]. Social value is obtained during this process when live-streaming consumers evaluate the reactions of other viewers, and if a brand would be considered acceptable among their social networks [11]. Prior literature has found a positive influence of social value on consumers’ purchasing behaviour [119,120]. In the live-streaming context, Yu and Zheng [36] found that consumers’ social status and personality could be highlighted in live-streaming shopping, further stimulating their purchase intention. Wongkitrungrueng and Assarut [11] also suggest that live-streaming customers could assign certain social symbolic meanings to their purchase process and use it to guide their consumption behaviours. Therefore, this study hypothesises:

H2: Social value positively influences customers’ purchase intention in live-streaming e-commerce.

### 3.3 Emotional value and purchase intention

Emotional value is the ability to stimulate consumers’ affective feelings or emotional states [79,121]. Mirror in the live-streaming e-commerce context, the emotional value reflects the ability of live-streaming e-commerce to stir up the viewers’ feelings or affective states [1]. Live-streaming’s interactive feature enables retailers to arouse viewers’ emotional pleasure in various ways [11]. For example, viewers could be entertained by participating in streamers’ online games or feel excited if they earn a special gift during the live-streaming [3]. This entertainment and excitement can be the core in forming a positive emotional feeling that drives people to use and purchase in live-streaming e-commerce. Guo et al. [1] stated that live-streaming e-commerce could create a sense of enjoyment and excitement, and these emotional feelings will significantly drive customers’ purchase intention. Hou et al. [3] also demonstrated that viewers could perceive emotional affection from the entertaining usage experience of live-streaming e-commerce and eventually shape their consuming behaviour. Therefore, we posit:

H3: Emotional value positively influences customers’ purchase intention in live-streaming e-commerce.

### 3.4 Epistemic value and purchase intention

Epistemic value is related to consumers’ variety and novelty-pursuing ideas or needs for knowledge that a particular merchandise satisfies [122]. Streamers constantly introduce interesting products with novel designs and characteristics that fulfil viewers’ variety and novelty-seeking intentions [3]. Meanwhile, facilitated by the real-time interactive feature of live-streaming, viewers’ can gain more comprehensive knowledge about the products by bringing up questions and doubts or asking streamers to introduce the product in their preferred way. Previous IS research has found a positive connection between epistemic value and consumers’ behaviours [123,124]. Qian et al. [125] also demonstrated that epistemic value in live-streaming service would significantly influence viewers’ purchase intention and engagement. To this end, this study proposes:

H4: Epistemic value positively influences customers’ purchase intention in live-streaming e-commerce.

### 3.5 Conditional value and purchase intention

Conditional value is perceived when a product or service meets consumers’ demands in specific context settings [126]. Conditional value in live-streaming e-commerce is well perceived by consumers, when streamers give discounts or coupons during their live shows and claim these promotions are only given to viewers watching at this moment. In addition, when celebrity streamers are involved in live-streaming, viewers can acquire extra conditional value from the interaction and engagement with those celebrities [127]. Although only limited studies have explored conditional value in the live-streaming e-commerce context, previous literature has demonstrated that conditional value has a significant influence on purchase behaviour in online service [81], mobile applications [121], or online games [128]. Therefore, we hypothesise:

H5: Conditional value positively influences customers’ purchase intention in live-streaming e-commerce.

### 3.6 Self-gratification value and purchase intention

Live-streaming e-commerce nowadays is not just a platform for online shopping. With the rapid development of technology and online business environment, various functions are developed and embedded in the live-streaming that have significantly enriched viewers’ usage experience [129]. Purchasing a certain product is no longer the only reason consumers watch live-streaming e-commerce. It also acts as a release for consumers to escape from stress at work and an entertaining way for viewers [130]. Watching and purchasing from live-streaming e-commerce has gradually become an indispensable part of daily life. Therefore, this study introduces a new independent variable, self-gratification value, to provide a more comprehensive understanding of live-streaming viewers’ perceived value. Self-gratification value is defined as the improvement in individual welfare after consuming a product or service, such as a release from pressure, diversion from a bad mood, or reduction of loneliness [92,131]. Consumers with a self-gratified attitude are reasonably more willing and spontaneous to purchase from live-streaming e-commerce. Previous studies also affirmed a positive relationship between self-gratification value and customer behaviours [62,92,131]. Therefore, this study posits:

H6: Self-gratification value positively influences customers’ purchase intention in live-streaming e-commerce.

### 3.7 Moderating effect of streamer popularity

Another research objective of this study is to investigate streamer popularity’s moderating effect on the relationship between consumption values and purchase intention. Although only limited studies have shed light on this specific interaction [106,132], previous literature has found that the endorsers’ popularity is a relevant construct in determining consumers’ attitudes and behaviours [1,106]. Kay et al. [133] suggested that influencers’ social media posts with more Likes will result in consumers’ higher purchase intention toward the endorsed products. Ladhari et al. [98] and Hill et al. [134] also demonstrated that consumers’ purchase behaviour in endorsed products is positively related to video bloggers’ popularity. In this study, streamers who enjoy high popularity and celebrity standing can easily capture millions of followers’ attention and spread persuasive information during live-streaming. In this way, viewers’ purchase intention developed from previous value perceptions could be fortified and stimulated by seeing their target product endorsed by a celebrity. For example, consumers’ trust in a celebrity endorser could be transferred into their trust in the functional performance of the endorsed products [25]. Viewers’ emotional feelings could be greatly aroused by interaction with popular celebrities and streamers [116]. Meanwhile, a popular streamer’s opinion will act as a bandwagon cue, leading to the social collective choice and belief formulated within their followers [135]. As a result, the commodities endorsed by popular streams are more likely to be accepted by numerous consumers, which in turn fortifies their influence [104]. Therefore, it is reasonable to postulate that different consumption values’ impact on purchase intention would be more significant for streamers with higher popularity, as proposed in H7(a, b, c, d, e, f):

H7: Streamers’ popularity strengthens the influence of functional value (H7a), social value (H7b), emotional value (H7c), epistemic value (H7d), conditional value (H7e), and self-gratification value (H7f) on customers’ purchase intention in live-streaming e-commerce.

## 4. Research method

### 4.1 Data collection

This study chooses users of several live-streaming e-commerce platforms as the target sample group to generate generalisable results. We specifically consider Taobao, Douyin, Kuaishou, Jindong and Pinduoduo. Among them, Taobao, Jingdong and Pinduoduo are typically known as the three leading e-commerce platforms in China, each with a vast consumer base and having successfully integrated live-streaming method into their e-commerce platforms. While Douyin and Kuaishou are two social media platforms that accumulate millions of customers by offering short video content. They have recently become two major forces in China’s live-streaming e-commerce area by adding live-streaming e-commerce function to their social media application [136]. All these platforms offered similar live-streaming e-commerce shopping experiences, and viewers’ purchase intention is unlikely to be cross-influenced by their shopping experience with alternative platforms [18]. Therefore, our study investigates how live-streaming e-commerce, in general, can influence viewers’ purchase intention.

The research data is collected by using the survey questionnaire method. Since the initial questionnaire was designed in English, a Chinese professor who majored in English was asked to perform the back-to-back translation. Then the Chinese version was further inspected by two experts in marketing and sent to five live-streaming e-commerce users with more than two years’ related shopping experience. Several changes were made to improve the language expression according to the suggestions. Before the final data collection, we assessed all the reliability and validity by conducting a pilot study among 30 respondents. The pilot study results indicate good reliability and validity of all the research variables. Finally, the survey questionnaire was generated on the Wenjuanxing website (http://www.wjx.cn), which has functions similar to Google Forms and is China’s most popular and professional data collection website. To increase the response rate and research validity, we chose the extra sample service offered by Wenjuanxing and paid CNY 4 for each valid response. This service could help researchers to randomly deliver the questionnaire to relevant and serious respondents. To further confirm the qualification of respondents, we added a screening question of, “Have you ever watched and purchased anything from live-streaming e-commerce recently?” only those respondents who answered yes were provided permission to the rest questionnaire. The respondents were then asked to recall their recent live-streaming e-commerce purchasing experience and a familiar streamer before answering the questions. Finally, a total of 590 questionnaires were distributed through the Wenjuanxing website from April 10 to July 15, 2022. After deleting the invalid surveys based on an inconvincible time frame to complete the questionnaire and identical answers to all the items, 457 responses were recorded as valid for final data analysis. Based on the 20:1 sample-to-variable ratio suggested by Hair et al. [137], our study has eight constructs which require a sample size > 160. Therefore, a sample size of 457 is appropriate to provide a valid structural model analysis.

Table 2 illustrates the demographic information of all 457 respondents. Most respondents are female (67.83%), 42.34% of the age between 18–35. A majority of respondents (26.70%) are students, and 38.07% claimed their monthly income is less than 3,000 CNY. The demographic information also indicates that Taobao Live is the most popular live-streaming e-commerce platform with 56.46% respondents, followed by Douyin and Kuaishou, respectively, with 24.73% and 7.88% of respondents. The sample’s proportion in each live-streaming e-commerce platform is shown to be correspondent with their market share [15]. Therefore, it can be concluded that the sample has good representativeness of the whole research population.

**Table 2:** Demographics information.

	Measure	Value	Percentage
Gender	Male	147	32.17%
	Female	310	67.83%
Age	Under 18	3	0.01%
	18–35	193	42.34%
	35–50	121	26.53%
	Above 50	140	31.12%
Occupation	Private Sector	93	20.35%
	Public Sector	82	17.94%
	Student	122	26.70%
	Retired	104	22.76%
	Self-Employment	56	12.25%
Monthly Income (CNY)	Less than 3,000	174	38.07%
	3,000–5,000	152	33.26%
	5,000–10,000	97	21.23%
	Above 10,000	34	7.44%
Preferred Live Streaming Platform	Taobao	258	56.46%
	Douyin	113	24.73%
	Kuaishou	36	7.88%
	Jingdong, Pinduoduo	50	10.94%

### 4.2 Measurement scales

The final research questionnaire consists of 33 items measuring eight constructs in the proposed model. All these items were adapted from previous literature to suit the live streaming context. The functional value items are adapted from Guo et al. [1] and Wongkitrungrueng and Assarut [11]. The items of social value are adapted from Singh et al. [16] and Wongkitrungrueng and Assarut [11]. The items of emotional value are adapted from Guo et al. [1] and Singh et al. [16]. The items of epistemic value are adapted from Assarut and Eiamkanchanalai [138] and Kaur et al. [139]. The items of conditional value are adapted from Hsieh et al. [140] and Yoon et al. [127]. The items of self-gratification value are adapted from El-Adly and Eid [141]. The items of purchase intention are adapted from Chen et al. [142]. Finally, the items of streamer popularity are adapted from Ladhari et al. [98]. Appendix A presents the details of all the measurement items. We survey these items by applying a seven-point Likert scale, where 1 = “strongly disagree” and 7 = “strongly agree”. Five control variables were also adopted in the research model to ensure the findings’ validity, including gender, age, occupation, income and preferred live-streaming platform.

### 4.3 Common method bias

This study adopted several remedies to mitigate common method bias according to Hulland et al.’s [143] method. First, the independent, dependent, and moderating variables are positioned arbitrarily at different places of the questionnaire to reduce sequential effects and in case a respondent perceives a causal relationship. In addition, we conducted Harman’s single factor test [144] and the result shows 37.483% of the variance accounted by a single factor, lower than the 50% criterion [145]. We further evaluated the variance inflation factor (VIF) and obtained the VIF values of all the constructs lie between 1.862–2.995. Based on Kock [146], VIF value lower than 3.3 demonstrates that collinearity is not a major concern in the research model.

## 5. Results

This study uses structural equation modelling (SEM) to assess the research model. The partial least square SEM (PLS-SEM) approach is adopted rather than covariance-based SEM (CB-SEM), because this is an exploratory study that aims at extending an existing structural theory and predicting several key target constructs [147]. We further applied SPSS 26 and SmartPLS 3.3.3 to conduct the measurement and structural model assessment.

### 5.1 Measurement model assessment

We applied confirmatory factor analysis (CFA) to establish the measurement model’s internal consistency reliability, convergent validity, and discriminant validity [147]. To verify the reliability and validity, we tested the items loading, composite reliability (CR), Cronbach’s alpha (α), and average variance extracted (AVE) of each research construct. As Table 3 shows, all the variables’ Cronbach’s α (ranging from 0.813 to 0.927) and CR (ranging from 0.862 to 0.943) have reached the criterion of 0.7 proposed by Hair et al. [147], denoting solid internal reliability.

**Table 3:** Reliability and validity.

Variables	No.	Loadings	(α)	CR	AVE
Functional Value	FV1	.800	0.826	0.879	0.594
	FV2	.796			
	FV3	.774			
	FV4	.862			
	FV5	.840			
Social Value	SV1	.757	0.813	0.862	0.574
	SV2	.745			
	SV3	.805			
	SV4	.838			
	SV5	.786			
Emotional Value	EMV1	.866	0.882	0.901	0.719
	EMV2	.842			
	EMV3	.884			
	EMV4	.845			
Epistemic Value	EPV1	.841	0.871	0.900	0.701
	EPV2	.912			
	EPV3	.888			
	EPV4	.794			
Conditional Value	CV1	.846	0.861	0.889	0.641
	CV2	.832			
	CV3	.799			
	CV4	.758			
	CV5	.806			
Self-Gratification Value	SGV1	.918	0.918	0.935	0.802
	SGV2	.939			
	SGV3	.923			
	SGV4	.891			
Purchase Intention	PI1	.886	0.859	0.904	0.779
	PI2	.904			
	PI3	.858			
Streamer Popularity	SP1	.892	0.927	0.943	0.824
	SP2	.918			
	SP3	.931			
	SP4	.899			

Next, we assess the convergent validity by applying item loading of 0.7, AVE of 0.5 as the criterion [147,148]. As illustrated in Table 3, all values of item loading (between 0.745 and 0.939) and AVE (between 0.574 and 0.824) have exceeded the target threshold, indicating adequate convergent validity.

Discriminant validity is assessed by comparing the square root of the AVE with the shared correlations between each pair of variables [149]. Table 4 shows that all diagonal values (lower left) are higher than the inter-construct correlations and thereby confirm the discriminant validity. The Heterotrait-Monotrait (HTMT) ratio is another approach to determining the discriminant validity. It is the ratio of the between-trait correlation to the within-trait correlation [150]. Table 4 shows that all variables’ HTMT ratios (upper right) were under the criterion of 0.85 [150], indicating adequate discriminant validity.

**Table 4:** Fornell-larcker criterion (lower left) & Heterotrait-Monotrait Ratio (HTMT).

	FV	SV	EMV	EPV	CV	SGV	PI	SP
Functional Value	**0.771**	0.394	0.636	0.403	0.684	0.557	0.457	0.392
Social Value	0.334	**0.757**	0.643	0.631	0.509	0.590	0.667	0.566
Emotional Value	0.572	0.613	**0.809**	0.612	0.639	0.667	0.727	0.608
Epistemic Value	0.463	0.548	0.610	**0.860**	0.716	0.584	0.686	0.395
Conditional Value	0.626	0.495	0.636	0.649	**0.801**	0.595	0.639	0.547
Self-Gratification Value	0.487	0.520	0.621	0.565	0.561	**0.918**	0.629	0.425
Purchase Intention	0.520	0.566	0.554	0.620	0.597	0.628	**0.883**	0.554
Streamer Popularity	0.355	0.446	0.478	0.218	0.407	0.365	0.466	**0.929**

**Note:** Bold numbers denote the square root of the AVE. The lower left values follow the Fornell–Larcker criterion, and the upper right values for the Heterotrait–monotrait criterion.

### 5.2 Structural model assessment

We then analyze the structural model to test the relationships among constructs. We first assessed the determinant coefficient R^2^ to evaluate the explanatory strength. Chinn [148] stated that in social science and business research, R^2^ of 0.190, 0.333, and 0.670 indicate a low, moderate and large explanatory strength. The test result shows that the R^2^ value of purchase intention is 0.692, implying the high prediction power of the research model. Next, we adopted the blindfolding technique and tested the Stone-Geisser *Q*^2^ to check the predictive relevance. The *Q*^2^ value of purchase intention is 0.525, indicating good prediction accuracy of the research model [151]. Furthermore, we also assessed the effect size (*f*^2^) based on the criterion provided by Cohen [152], that 0.35, 0.15, and 0.02 represent a large, moderate, and small effect. Table 5 presents the test outcomes of determinant coefficient (R^2^), predictive relevance(*Q*^2^) and effect size (*f*^2^).

**Table 5:** Determinant coefficient (R^2^), predictive relevance(*Q*^2^), and effect size (*f*^2^).

Exogenous Variable	Endogenous Variable	R^2^	*Q* ^2^	Effect Size (*f*^2^)	Decision
Functional Value	Purchase Intention	0.692	0.525	0.122	Small
Social Value				0.027	Small
Emotional Value				0.067	Small
Epistemic Value				0.001	No
Conditional Value				0.097	Small
Self-Gratification Value				0.083	Small

**Fig 2:**
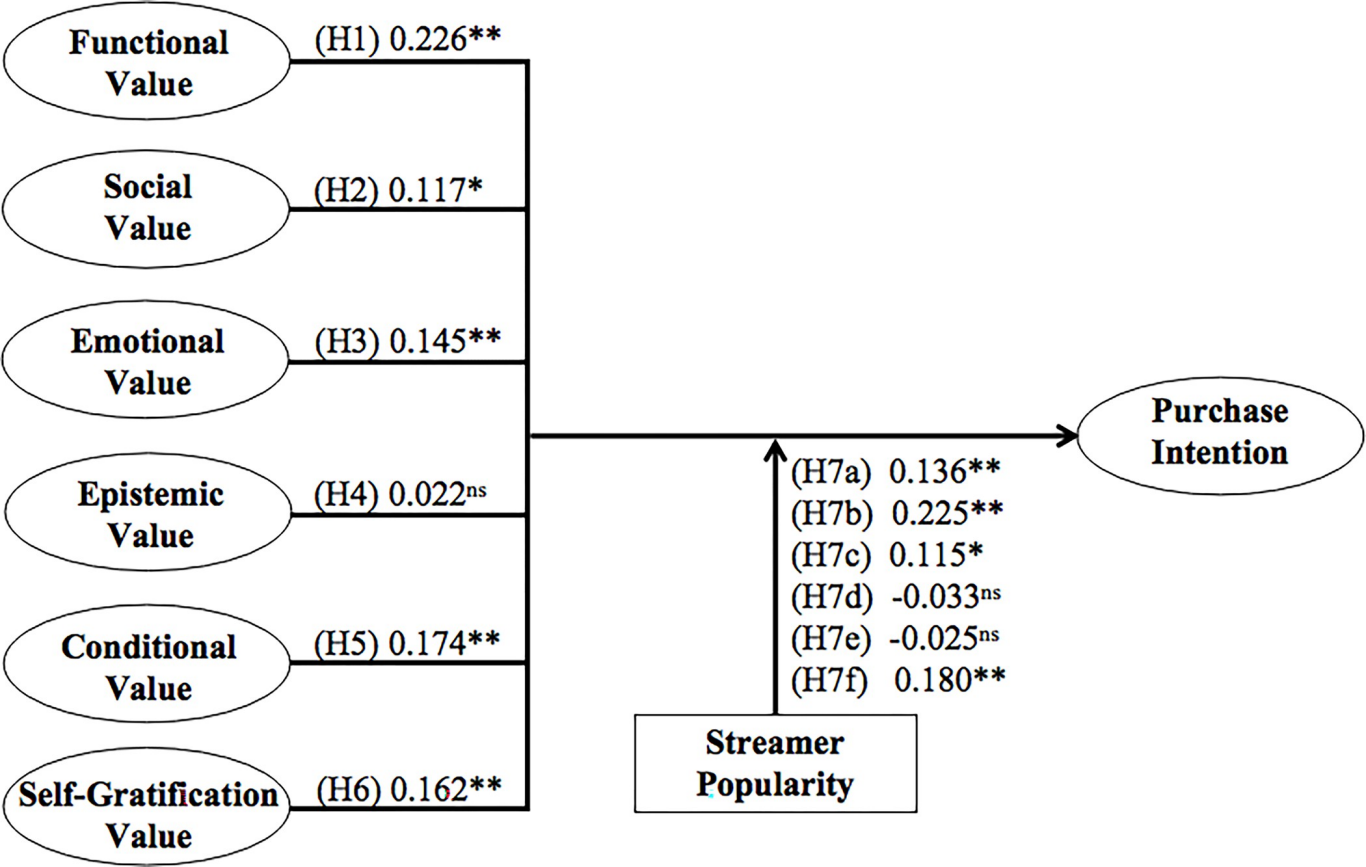
Structural model results. * *p* < 0.05, ** *p* < 0.01, *NS*: not significant.

We then calculate the path coefficient and T-values by using a bootstrapping subsampling technique (5,000 times) to test the research hypotheses. Tables 6 and 7, and Fig 2 present the output of SEM analysis. Regarding the direct relationships between consumption values and purchase intention, five of six hypotheses were supported by the result (Table 7). In specific, five of the consumption values are significant determinants of purchase intention, including functional (*β* = 0.226, *t* = 5.482, *p* = 0.000), social (*β* = 0.117, *t* = 2.210, *p* = 0.014), emotional (*β* = 0.145, *t* = 5.482, *p* = 0.000), conditional (*β* = 0.174, *t* = 4.410, *p* = 0.000) and self-gratification value (*β* = 0.162, *t* = 3.330, *p* = 0.000), supporting H1, H2, H3, H5 and H6. Meanwhile, epistemic value (*β* = 0.022, *t* = 0.544, *p* = 0.383) is not a significant driver of satisfaction, H4 is not supported.

**Table 6:** Direct effect.

	Path	(β)	ST DEV.	T-Values	Effect Size (*f*^2^)	Confidence Intervals		Decision
						Lower	Upper	
H1	FV→PI	0.226	0.054	5.482**	0.122	0.131	0.304	Supported
H2	SV→PI	0.117	0.036	2.210*	0.027	0.039	0.182	Supported
H3	EMV→PI	0.145	0.038	2.773**	0.067	0.071	0.240	Supported
H4	EPV→PI	0.022	0.035	0.544^ns^	0.001	-0.048	0.115	Not-Supported
H5	CV→PI	0.174	0.049	4.410**	0.097	0.110	0.240	Supported
H6	SGV→PI	0.162	0.038	3.330**	0.083	0.087	0.225	Supported

Note: * *p* < 0.05

** *p* < 0.01, *NS*: Not significant.

**Table 7:** Moderating effect.

No.	Path	Path Coef. (β)	ST DEV.	T-Values	Confidence Intervals		Decision
					5%	95%	
H7a	Moderating Effect of SP on FV→PI	0.136	0.043	3.185**	0.079	0.195	Supported
H7b	Moderating Effect of SP on SV→PI	0.225	0.054	4.167**	0.131	0.304	Supported
H7c	Moderating Effect of SP on EMV→PI	0.115	0.051	2.581*	0.027	0.218	Supported
H7d	Moderating Effect of SP on EPV→PI	-0.033	0.047	0.744^ns^	-0.084	0.012	Not-Supported
H7e	Moderating Effect of SP on CV→PI	-0.025	0.042	0.395^ns^	-0.135	0.109	Not-Supported
H7f	Moderating Effect of SP on SGV→PI	0.180	0.040	4.461**	0.111	0.256	Supported

Note: * *p* < 0.05

** *p* < 0.01, *NS*: Not significant.

**Fig 3:**
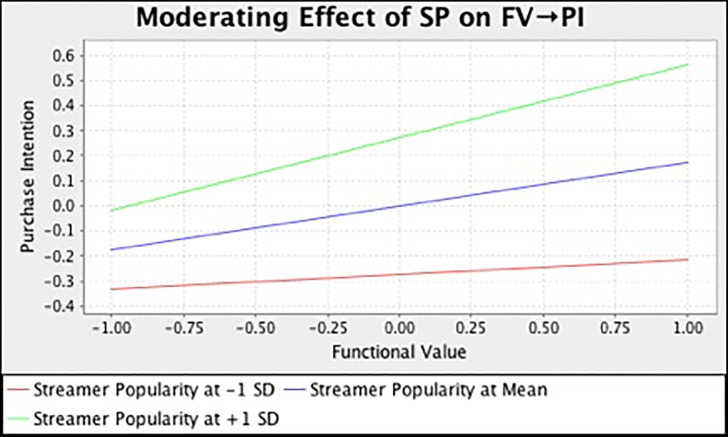
Moderating Effect of SP on FV → PI.

**Fig 4:**
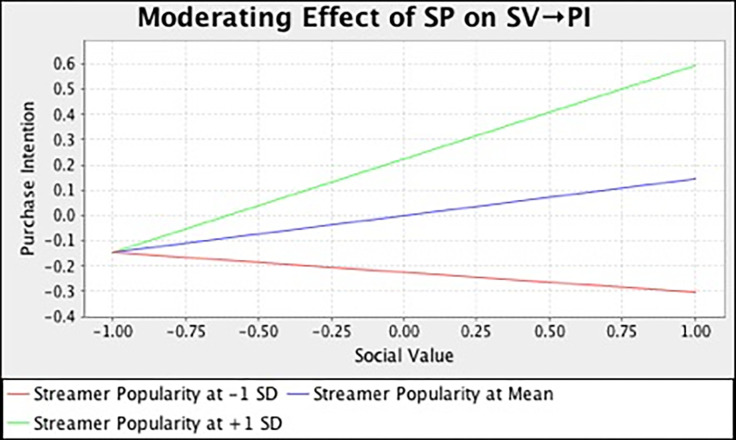
Moderating Effect of SP on SV → PI.

**Fig 5:**
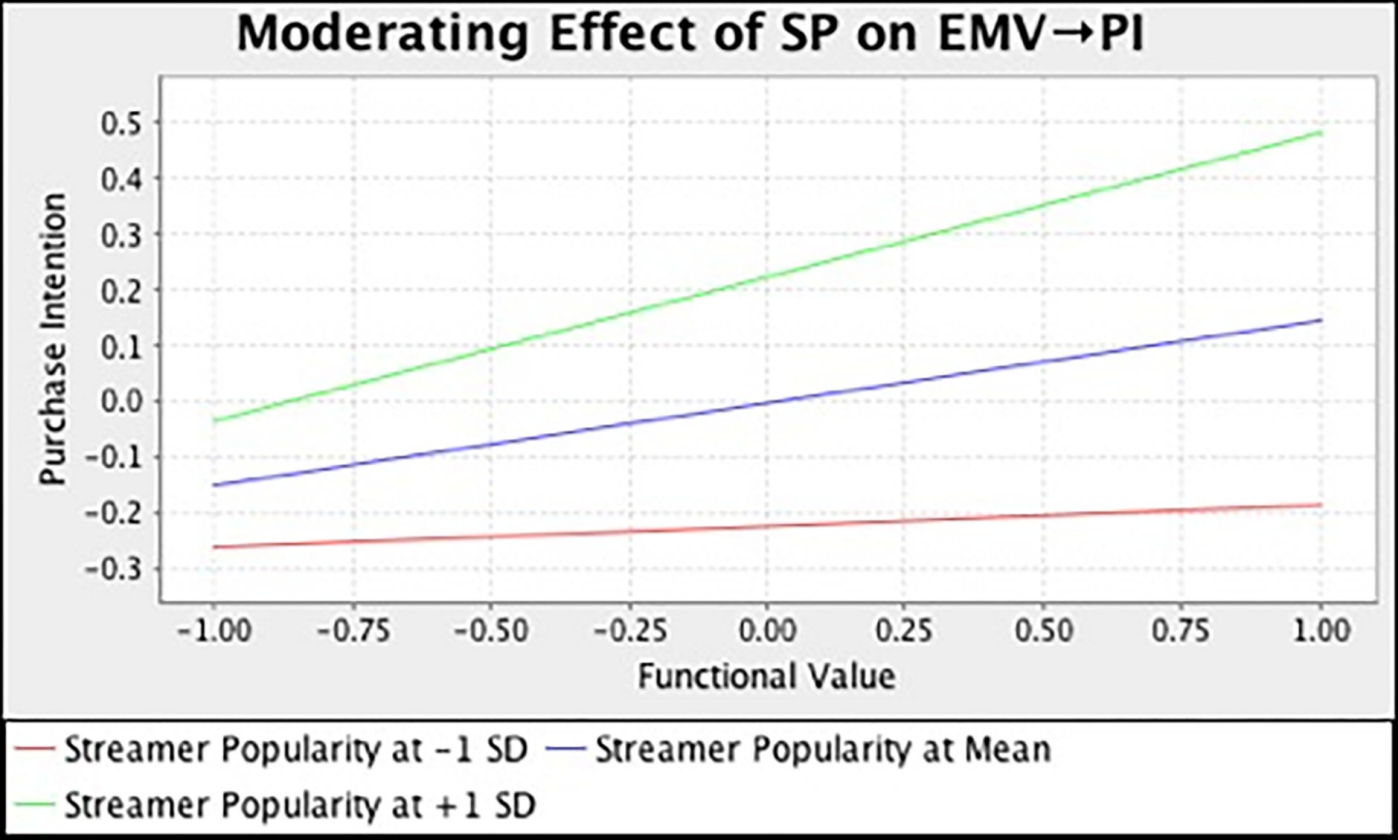
Moderating Effect of SP on EMV → PI.

**Fig 6:**
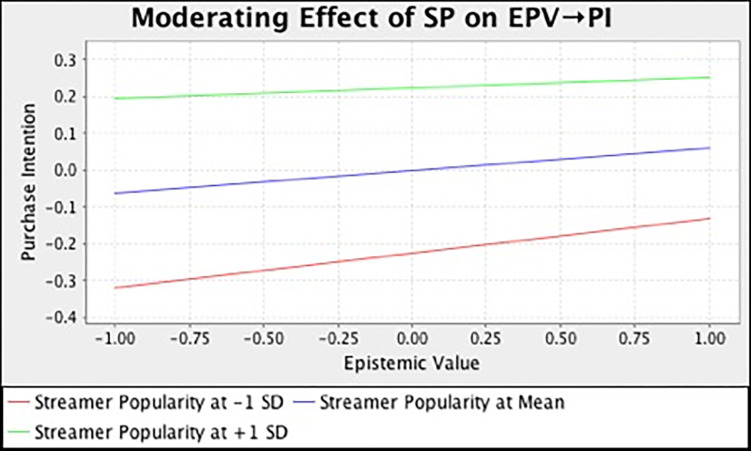
Moderating Effect of SP on EPV → PI.

**Fig 7:**
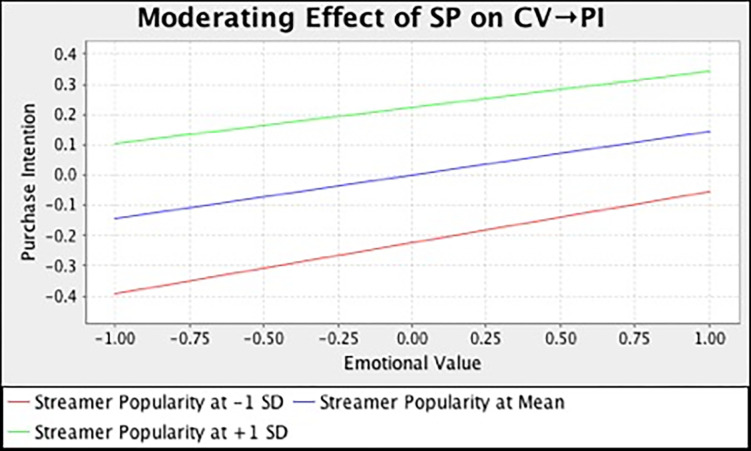
Moderating Effect of SP on CV → PI.

**Fig 8:**
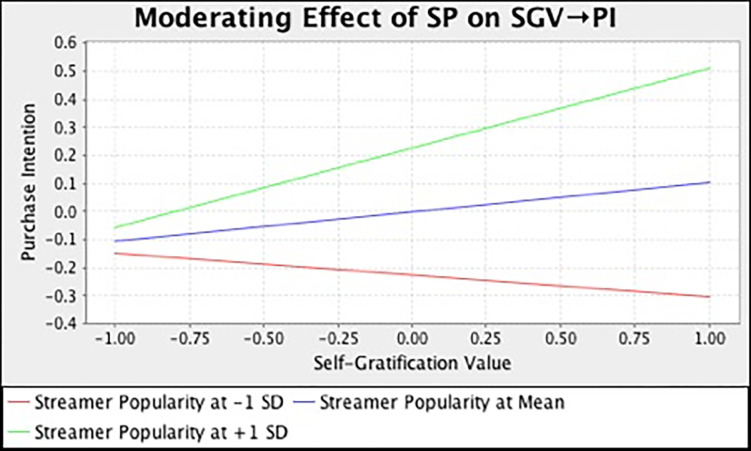
Moderating Effect of SP on SGV → PI.

In this study, we also examined the moderating effect of streamer popularity on the relationships between different consumption values and purchase intention. Table 7 and Fig 2 summarize the output of SEM analysis with bootstrapping technique. The result demonstrated that streamer popularity has strengthened the relationship between functional (*β* = 0.136, *t* = 3.185, *p* = 0.000), social (*β* = 0.225, *t* = 4.167, *p* = 0.000), emotional (*β* = 0.115, *t* = 2.581, *p* = 0.023), self-gratification (*β* = 0.180, *t* = 4.461, *p* = 0.000) value and purchase intention. However, streamer popularity does not moderate the influence of epistemic (*β* = -0.033, *t* = 0.744, *p* = 0.693) and conditional value (*β* = -0.025, *t* = 0.395, *p* = 0.530) on customers’ purchase intention. In the meantime, we also adopted the simple slope assessment to illustrate the moderating effect. Fig 3–8 present the results of simple slope analysis in Smart-PLS. As shown in Figs 3, 4, 5 and 8, which respectively represents functional, social, emotional, and self-gratification value, the green curve (higher streamer popularity group) is steeper than the red curve (lower streamer popularity group), means that positive relationship between functional, social, emotional, self-gratification value and purchase intention is stronger when streamer popularity is high, thus supporting H7a, H7b, H7c, and H7f. On the contrary, in Figs 6 and 7, the gradient of the green line (higher streamer popularity group) has no significant difference compared to the red line (lower streamer popularity group), implying no moderating effect of streamer popularity on the relationship between epistemic value, conditional value and purchase intention, H7d and H7e are not supported.

## 6. Discussion

The current study investigates the influencing factors of live-streaming e-commerce customers’ purchase intention and the moderating effect of streamer popularity. We designed a theoretical model based on the TCV and empirically examined the impact of functional, social, emotional, epistemic, conditional and self-gratification value on customers’ purchase intention in live-streaming e-commerce. This study also considered the moderating effect of streamer popularity on the relationship between different consumption values and purchase intention. Our study results indicate that five of six consumption values are positively related to purchase intention, and streamer popularity has strengthened the impact of four consumption values on purchase intention.

### 6.1 Consumption values and purchase intention

First, our results revealed that functional value is the primary determinant of customers’ purchase intention in live-streaming e-commerce. This finding is consistent with earlier studies in the live-streaming context, such as Yu and Zheng [36] and Guo et al. [116]. Functional value in live-streaming e-commerce is related to its unique functions and advantages in the product exhibition process [9]. Functional value could be well acquired by the customers when they have a more comprehensive view of the products, or when the streamer could immediately answer their specific doubts or questions. These customers whose expectations have been fulfilled and whose doubts have been answered are reasonably more willing to buy the products.

Our findings also show that social value is a significant driver of live-streaming e-commerce users’ purchase intention. This finding correlates with several previous live-streaming studies [11,36,120]. Live-streaming e-commerce is a social platform where users can share their ideas, opinions, and feedbacks during the exhibition [16]. On this platform, customers can assess the social popularity of the products among other viewers, communicate with them about their usage experience, or just talk about their shared interested topics. In this way, viewers’ social identity and personality could be reinforced, further stimulating their purchasing behaviour.

Next, we found that emotional value is also an important determinant of customers’ purchase intention. This echoes prior studies in similar live-streaming contexts, such as Guo et al. [1] and Hou et al. [3]. Emotional value in live streaming e-commerce stands for its ability to stimulate viewers’ positive feelings or affective states [1]. It is a significant motivational factor for users’ purchase intention [153]. Viewers’ emotional pleasures could be aroused by live-streaming in various ways, such as through the special gifts given by the streamer, or by participating in the online games and activities organized by the steamer. This emotional pleasure is further fortified by the streamer’s distinctive features, such as humorous character or physical attractiveness. As a result, the entertainment, excitement, and fulfilment perceived by viewers have laid the foundations for their emotional states of mind and will eventually drive their purchase intention.

However, the findings demonstrated an insignificant connection between epistemic value and purchase intention. This is an unexpected result and contradicts a number of previous studies [125,154,155]. There are two possible explanations for this contradiction. Firstly, studies that concluded a positive connection between epistemic value and customers behaviour were mostly carried out in contexts that closely related to epistemic value, such as in tourism [156] or food industry [154,155]. In these context settings, seeking novelty (new tourist spot) or variety (different food tastes) is the core of customers’ value expectations. However, this is not the case in live-streaming e-commerce. As an online shopping channel that has existed for some time, seeking novel experiences is no longer the primary concern of the viewers. Secondly, the essence of live-streaming e-commerce is about selling products [24]. Compared with epistemic value, viewers’ purchase intention is more likely triggered by the vivid exhibition of products’ physical performance (functional value) or positive peer comments (social value). Therefore, this study concluded epistemic value does not influence customers’ purchase intention in live-streaming e-commerce.

Conditional value is found to be a significant driver of purchase intention in live-streaming e-commerce. This finding is consistent with prior studies of Talwar et al. [81] and Chakraborty and Paul [121]. Conditional value stands for live-streaming e-commerce’s ability to satisfy viewers’ demands in specific circumstances [126]. It can be perceived by customers when they communicate with their favourite celebrity streamers, or when merchants offer conditional discounts only to those online viewers. As a result, viewers could buy their wanted product at an unparalleled low price and engage with their celebrity streamer during the shopping process. Moreover, during the COVID-19 pandemic, live-streaming e-commerce offers a unique conditional benefit, allowing people to shop in a virtually real environment without stepping out of the house. Subsequently, all these conditional values perceived by viewers would positively stimulate their purchase intention in live-streaming e-commerce.

Our study also demonstrated that self-gratification value is a significant determinant of purchase intention in live-streaming e-commerce. This finding is in line with several prior studies [92,131], and also provides solid justifications for extending the original TCV by incorporating self-gratification value as an extra independent variable in live-streaming e-commerce context. Self-gratification value is received by live-streaming viewers from the effectiveness of the overall watching and purchasing experience to reduce their tension and stress [62]. As a compound platform of video clips, real-time interaction, and online shopping [3,4], live-streaming e-commerce nowadays is becoming an important way for ordinary people to divert their attention from routine work and entertaining themselves [130]. When users feel self-gratified with the overall performance of live-streaming e-commerce, it is reasonable to predict they are more likely to watch and purchase in live-streaming e-commerce.

### 6.2 Moderating effect of streamer popularity

Although previous researchers have mentioned the direct influence of streamer popularity on customer behaviour, our research is among a few studies that have tested the interaction of streamer popularity with the connection between value and purchase intention. The result of the moderating effect analysis provides several interesting insights. First of all, as we expected, streamer popularity has strengthened the impact of functional, social, emotional and self-gratification value on live-streaming e-commerce viewers’ purchase intention, indicating that for streamers with higher popularity, viewers’ perceived functional, social, emotional and self-gratification are more likely to transit to their purchase intention. The possible explanation is that popular streamers are more likely to attract viewers’ attention and disseminate positive information about their endorsed products. Their tendentious promotional propaganda would sound more convincing and attractive to viewers than less popular streamers, subsequently increasing the impact of functional value on viewers’ purchase intention. Similarly, judgment from a popular streamer will influence the social collective choice and belief among viewers, the attachment to a popular streamer or the fans’ community will result in a stronger influence of viewers’ perceived social value on purchase intention. Next, popular streamers and celebrities could further arouse viewers’ emotional states and create an atmosphere of excitement, which is the cradle for impulse buying intention. Finally, popular streamers are typically with certain advantages in their physical attractiveness or outstanding speaking skills, and their live-stream program is deliberately designed as a delicate mixture of entertaining content and e-commerce purpose. Viewers immersed in such live-streaming rooms tend to be more gratified and feel obligated to buy something.

On the contrary, our findings show that streamer popularity does not strengthen the influence of epistemic and conditional value on customers’ purchase intention. We provide two possible explanations for these unexpected findings. First, epistemic value in live-streaming e-commerce is mainly reflected by searching for novelty products or experiences. However, popular streamers tend to choose traditional products with stable quality and performance to avoid risks in after-sales and protect their reputation. As such, streamer popularity has only limited influence on epistemic value and purchase intention interaction. Secondly, conditional value is conceptually related to situational factors such as exclusive discounts or coupons offered during live streaming, and these situational factors’ influence does not rely on the streamer’s popularity. Viewers’ purchase intention for a certain product will always be triggered by a special discount offered, regardless of whether the discounts or coupons are given by a popular streamer or not.

### 6.3 Theoretical implications

This study has three main theoretical contributions. First, our research is among limited studies that examine live-streaming e-commerce customers’ behaviour from a value perspective. This study provides a validated comprehensive multi-dimensional value framework in live-streaming e-commerce context. The study findings demonstrated that five of six consumption values are significant determinants of customers’ purchase intention in live-streaming e-commerce. Secondly, we expand the original Theory of Consumption Values by introducing self-gratification value as a new contextual independent variable. The empirical results suggest that self-gratification value is a significant driver of customers’ purchase intention in live-streaming e-commerce. Thus, this study has expanded the usage of TCV and strengthened its explaining power. It also demonstrates that TCV can be further developed and adapted to various research contexts to better understand consumer behaviour. Thirdly, this study incorporates a moderating variable streamer popularity into the research framework and tests its moderating effect on the relationship between consumption values and purchase intention. The empirical study results show that streamer popularity has strengthened the impact of four consumption values on purchase intention. As such, this study provides useful insights into the celebrity streamers’ moderating mechanism underlying the link between different consumption values and purchase intention.

### 6.4 Practical implications

Our findings also have several practical contributions for live-streaming e-commerce merchants and operators. First, this study offers insights into purchase intention in live-streaming e-commerce from a customer’s value perspective. Merchants and operators can use these findings to strengthen customers’ value perceptions and stimulate their purchase intention. They should prioritize company resources and optimize marketing strategies to provide services that could deliver functional, social, emotional, conditional, and self-gratification value. For example, merchants could make the product exhibition process more informative and attractive to increase functional value. They can also intentionally guide viewers to participate in online discussions to cultivate their social value, or provide more exciting online games and entertaining content to stimulate customers’ emotional and self-gratification value.

In addition, merchants and operators need to understand that customers’ purchase intention will not be driven by epistemic value. They should realize that although live-streaming e-commerce has been widely discussed as an emerging technological trend that provides a novel shopping experience and creates massive customer engagement [6,11], the fact is that viewers will not buy from live-streaming only because their curiosity and variety-seeking intentions have been fulfilled. Therefore, merchants should avoid putting too much effort into epistemic value initiatives.

Furthermore, merchants need to be aware of the moderating role of streamer popularity and wield it cautiously. For online stores or products that have a tight connection with functional, social, emotional, and self-gratification value, such as home appliances (functional), wine (social), games (emotional) or music (self-gratification), merchants could increase their sales by inviting famous celebrities or streamers to host their live-stream shows. On the contrary, for those online stores or products closely related to epistemic (tourism agency) and conditional value (outlets), investing extra money in popular streamers is not recommended.

## 7. Limitations and future research

Although this study has several sound theoretical and practical contributions, we acknowledge several limitations. First of all, we collect data from users of several Chinese live-streaming platforms. Future studies can be performed in different countries and cultural backgrounds to increase the generalizability of the findings. Second, although we include several control variables in our paper to give a general finding, we hadn’t considered different product types that may influence purchase intention. It is recommended for future studies to explore the impact of other product-related elements on live-streaming purchase intention. Third, our study examines purchase intention from a consumer’s angle, while platforms and merchants are also indispensable parts of live-streaming e-commerce [11]. Future research can try to explain consumer behaviour by incorporating diversified perspectives. Lastly, this is a cross-sectional study that only could explain customer behaviour at a specific point in time. Future studies may use longitudinal research to explore possible changes in live-streaming e-commerce users’ purchase intention.

## Supporting information

S1 Data Original empirical study data. (XLSX)

S1 Appendix A constructs, measurement items and sources. (DOCX)
