# Supplementary material for: Consumers purchase intention in live-streaming e-commerce: A consumption value perspective and the role of streamer popularity
Source: PLoS One. 2024 Feb 15;19(2):e0296339. doi: 10.1371/journal.pone.0296339 (PMC10868799; doi:10.1371/journal.pone.0296339)
Supplement: S1 Appendix — (DOCX) [file pone.0296339.s002.docx]

**Appendix A**

Constructs, Measurement Items and Sources

| **Constructs** | **No.** | **Measurement Items** | **Sources** |
| --- | --- | --- | --- |
| Functional Value | FV1 | The way a product is presented in this streamer’s live-streaming room (e.g., a seller’s try-on) helps me to visualize the how a product works on a real figure. | (Guo et al., 2022; Wongkitrungrueng & Assarut, 2020) |
|  | FV2 | The introduction of streamer and interactive function help me find the right product I want. |  |
|  | FV3 | The way this streamer presents a product gives me as much sensory information as I would experience in a store. |  |
|  | FV4 | I can accomplish my shopping goals in this streamer’s live-streaming room. |  |
|  | FV5 | Live-streaming provides more information I need to make a purchase decision. |  |
| Social Value | SV1 | I can find products that fit my style and social identity in this streamer’s live-streaming room. | (Singh et al., 2021; Wongkitrungrueng & Assarut, 2020) |
|  | SV2 | I feel that I belong to the customer segment of this streamer. |  |
|  | SV3 | I can infer social acceptance from other customers’ comments and interactions with the streamer during the live stream. |  |
|  | SV4 | I value live-streaming e-commerce because they are popular among my social connections. |  |
|  | SV5 | I feel trendy and eager to share my live shopping experience to my friends/acquaintances. |  |
| Emotional Value | EMV1 | This streamer’s live-streaming program gives me a sense of happiness and fun, not just because I am able to purchase the products I want. | (Guo et al., 2022; Singh et al., 2021) |
|  | EMV2 | I feel excited after watching or shopping through live-streaming e-commerce. |  |
|  | EMV3 | I enjoyed the experience of watching and shopping through live-streaming e-commerce. |  |
|  | EMV4 | I feel much better after using live-streaming e-commerce. |  |
| Epistemic Value | EPV1 | I use live-streaming e-commerce to see what are new and interesting products available. | (Assarut & Eiamkanchanalai, 2015; Kaur et al., 2018) |
|  | EPV2 | I use live-streaming e-commerce to keep up with new fashion and trends. |  |
|  | EPV3 | Live-streaming e-commerce arouses me with some interesting knowledge or novel using tricks about the products. |  |
|  | EPV4 | Live-streaming e-commerce arouses and satisfies my curiosity by providing a novel buying experience. |  |
| Conditional Value | CV1 | I value the real-time information and interaction that live-streaming e-commerce makes possible. | (Hsieh et al., 2021; Yoon et al., 2021) |
|  | CV2 | Live-streaming e-commerce offers a competitive price, extra discounts and frequent promotions for the products. |  |
|  | CV3 | I like to see and interact with certain streamers in live-streaming e-commerce. |  |
|  | CV4 | I like to spend time and engage in the followers’ community within the live-streaming e-commerce. |  |
|  | CV5 | I can enjoy shopping at home without going out in live-streaming e-commerce. |  |
| Self-Gratification Value | SGV1 | I feel it is an escape from life pressure when I watch live-streaming e-commerce. | (El-Adly & Eid, 2017) |
|  | SGV2 | I can forget my problems when I watching and shopping from live-streaming e-commerce. |  |
|  | SGV3 | Usage experience of live-streaming e-commerce helped me release stress and relax. |  |
|  | SGV4 | For me, live-streaming is a helpful way to do something different from my daily routine. |  |
| Purchase Intention | PI1 | I will consider live streaming e-commerce as my first shopping choice. | (Chen et al., 2017) |
|  | PI2 | I intend to purchase products or services through live streaming e-commerce. |  |
|  | PI3 | I expect that I will purchase products or services through live streaming e-commerce. |  |
| Streamer Popularity | SP1 | My favorite streamer is famous. | (Ladhari et al., 2020) |
|  | SP2 | My favorite streamer has a lot of followers. |  |
|  | SP3 | The popularity of my favorite streamer is increasing. |  |
|  | SP4 | My favorite streamer has a lot of viewers each time he/she streams. |  |
